# Synthesis and Study of SrTiO_3_/TiO_2_ Hybrid Perovskite Nanotubes by Electrochemical Anodization

**DOI:** 10.3390/molecules29051101

**Published:** 2024-02-29

**Authors:** Madina Bissenova, Arman Umirzakov, Konstantin Mit, Almaz Mereke, Yerlan Yerubayev, Aigerim Serik, Zhengisbek Kuspanov

**Affiliations:** 1 Institute of Physics and Technology, Almaty 050032, Kazakhstan; m-bisenova@list.ru (M.B.); arman_umirzakov@mail.ru (A.U.); konstantin-mit@yandex.ru (K.M.);; 2 Institute of Nuclear Physics, Almaty 050032, Kazakhstan; aigerimserik3508@gmail.com; 3 Department of Materials Science, Nanotechnology and Engineering Physics, Satbaev University, Almaty 050032, Kazakhstan; 4 Department of Mechanics and Mechanical Engineering, M.Kh. Dulaty Taraz Regional University, Taraz 080000, Kazakhstan

**Keywords:** photocatalyst, TiO_2_, TNT, SrTiO_3_, anodizing

## Abstract

Layers of TiO_2_ nanotubes formed by the anodization process represent an area of active research in the context of innovative energy conversion and storage systems. Titanium nanotubes (TNTs) have attracted attention because of their unique properties, especially their high surface-to-volume ratio, which makes them a desirable material for various technological applications. The anodization method is widely used to produce TNTs because of its simplicity and relative cheapness; the method enables precise control over the thickness of TiO_2_ nanotubes. Anodization can also be used to create decorative and colored coatings on titanium nanotubes. In this study, a combined structure including anodic TiO_2_ nanotubes and SrTiO_3_ particles was fabricated using chemical synthesis techniques. TiO_2_ nanotubes were prepared by anodizing them in ethylene glycol containing NH_4_F and H_2_O while applying a voltage of 30 volts. An anode nanotube array heat-treated at 450 °C was then placed in an autoclave filled with dilute SrTiO_3_ solution. Scanning electron microscopy (SEM) analysis showed that the TNTs were characterized by clear and open tube ends, with an average outer diameter of 1.01 μm and an inner diameter of 69 nm, and their length is 133 nm. The results confirm the successful formation of a structure that can be potentially applied in a variety of applications, including hydrogen production by the photocatalytic decomposition of water under sunlight.

## 1. Introduction

Rapid growth in the world population has increased the demand for energy, the bulk of which is provided by fossil fuels for power generation, industrial needs, and transportation [1,2,3,4]. However, in addition to limited availability, the use of fossil fuels has a negative impact on the environment, creating by-products such as carbon, nitrogen, and sulfur oxides [5,6]. Therefore, there is an urgent need to develop cleaner alternative energy sources that are sustainable and have a minimal impact on the environment [7,8]. Hydrogen stands out as a clean and efficient energy source, and its production is becoming an important challenge in the field of sustainable energy sources.

Water is an abundant source of hydrogen, but given the need to introduce energy to overcome the energy barrier associated with chemical stability, it is difficult to separate water into stoichiometric hydrogen and oxygen on an industrial scale. Nevertheless, one method of hydrogen production is the photocatalytic splitting of water. Photocatalysis can efficiently utilize solar energy to split water into its individual elements [9,10,11]. This is a unique and promising method of hydrogen production based on the use of solar energy to convert water into hydrogen and oxygen [12]. This process could be an important step toward sustainable energy sources, as it combines the efficiency of solar panels with the ability to produce clean hydrogen. In particular, photocatalysis has been shown to be a more efficient form of wastewater treatment because of the impressive efficiency of photocatalytic removal, rapid oxidation process, lower costs, and a lack of toxicity [13]. Photocatalytic water splitting involves the use of semiconductors as photocatalysts. The most studied photocatalysts are TiO_2_, ZnO, CdS, and SrTiO_3_, which are used for various photocatalytic applications including photocatalytic water splitting [14,15,16]. To achieve efficient photocatalytic water splitting, a sophisticated photocatalyst is required that can overcome problems in the water oxidation process.

Titanium dioxide (titanium, TiO_2_) is considered the most promising and versatile material. Over the past decades, TiO_2_ has been extensively investigated in various fields because of its unique properties such as outstanding corrosion resistance, high biocompatibility, suitable bandgap for water splitting, and stable physicochemical characteristics [17,18]. The narrow– bandgap facilitates the more efficient collection of solar energy, making it an ideal material for creating an electron–hole pair. This pair is actively involved in redox reactions and finds applications in various fields, such as dyes, food, biomedicine, photocatalysis, photodegradation of water, photosensitive materials, dye-sensitized solar cells, and gas-sensitive devices. In recent years, significant research efforts have been devoted to the development of new nanomaterials, including nanostructured titanium obtained via anodization, sol–gel, hydrothermal treatment, and vapor deposition techniques [19]. Nowadays, a wide range of materials are required to develop and research advanced devices suitable for various commercial applications. Nanomaterials play a key role in emerging technologies, enabling the creation of high-performance devices [20,21]. The performance of such devices is largely determined by the geometry, shape, and morphology of the nanostructures [7]. The exponential growth in the literature indicates that interest in the nanoscale began in the 1990s. Interest in the nanoscale is driven by the commercial availability of tools used to manipulate and measure nanoscale characteristics for several reasons: (1) the anticipation of the novel physical, chemical, and biological properties of nanostructures; (2) the assumption that nanostructures will provide new building blocks for innovative materials with unique properties; (3) the miniaturization of the semiconductor industry to the nanoscale; and (4) the recognition that molecular mechanisms in biological cells function at the nanoscale [22].

## 2. Results and Discussion

**Figure 1:**
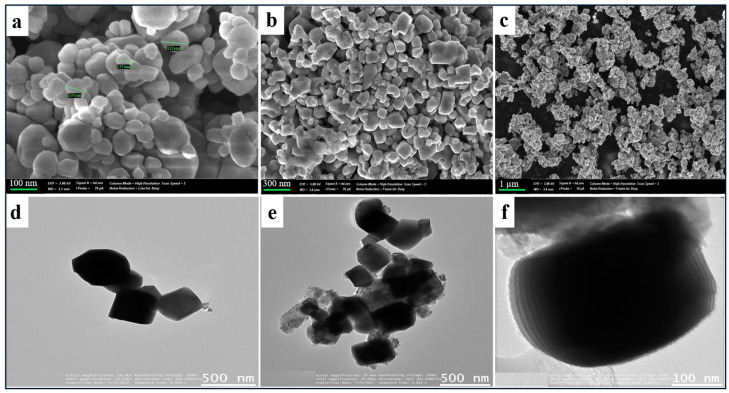
SEM (**a**–**c**) and TEM (**d**–**f**) images at different magnifications of cubic SrTiO_3_ obtained by a chemical precipitation method.

The morphology of the obtained SrTiO_3_ samples was studied using scanning (SEM) and transmission (TEM) electron microscopes at different resolutions. The scanning electron microscopy results (Figure 1a–c) show that the SrTiO_3_ particles that calcined at 900 °C possess cubic shapes and have sizes ranging from 150 nm to 300 nm. Calcination at 800 °C leads to the formation of finer particles but with more significant numbers of impurities such as SrCO_3_. Based on the literature and experimental data [23,24], the optimal calcination temperature is 900 °C, which is followed by treatment in 1 m nitric acid solution to remove residual SrCO_3_. However, it is worth noting that the particle sizes are highly heterogeneous. Given studies in the literature, doping SrTiO_3_ with other elements, such as Al or Mn, can contribute to the size reduction and distortion in SrTiO_3_‘s crystal shape.

In the case of TEM, clearly formed cubes of SrTiO_3_ with anisotropic structures with an average size of about 200 nm are clearly visible, as shown in Figure 1d–f. An important feature of these particles is the anisotropic structure, which creates a difference in energy at different faces, leading to the formation of p–n junctions. This allows the charge within each photocatalyst particle to be separated using an inter-domain electric field. Thus, electrons are concentrated on some faces and holes on other faces, which provides for the separation of photocatalytic reduction and oxidation processes on different faces. Given the anisotropic crystal structure, the selective deposition of catalysts takes place, which leads to the release of hydrogen and oxygen on the faces of the cubic photocatalyst.

**Figure 2:**
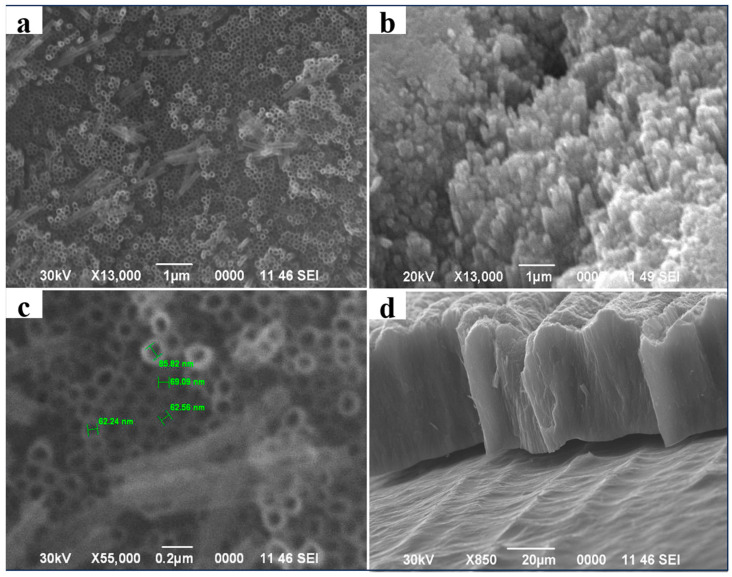
(**a**) SEM images showing the top surface of the TNT anode array; (**b**) the TNT@SrTiO_3_ array; (**c**) the top view and (**d**) side view of samples with magnification of the surface of the anode array prepared at 30 V.

Among various nanostructured oxide materials, TiO_2_ nanotubes have been emphasized because of their improved properties, economical design, and higher surface-to-volume ratio [25]. TNTs with high specific surface areas, ion exchange abilities, and photocatalytic properties have been considered for various potential applications and can be excellent candidates as catalysts in photocatalysis [26]. Figure 2 shows images of the top surfaces of the anodized TiO_2_ nanotube samples before and after the deposition of SrTiO_3_ on their surfaces. The top surfaces of the anodized and annealed TNTs at 450 °C shown in Figure 2a show well-defined tubes with open ends that form a hexagonal order. This is typical of anodization, as previously noted in [27]. After applying SrTiO_3_, pronounced morphological changes are observed with the presence of interface regions between SrTiO_3_ and TNT, which indicates the success of the combination (Figure 2b). During 6 h of autoclave treatment, the surface showed a tendency to be coated with nanoparticles, and uneven deposition was also found. Agglomerates are formed on the surface, and round holes corresponding to TNT are still visible. Note that increasing the treatment time to 6 h significantly affects the surface morphology, leading to the formation of larger agglomerates and the blocking of the tube tops [28]. Figure 2c shows that the initial TNTs have an average outer diameter of 1 μm, while Figure 2a shows an inner diameter of 69 nm. The length of the tubes is 133 nm, as seen in the inset. A cross-sectional view of a freestanding titanium dioxide membrane with an average thickness of more than 50 nm is shown in Figure 2d, mechanically collapsed for visualization. In a related study [25], Paulose et al. obtained nanotubes measuring 360 μm in length over a 96 h period, utilizing a voltage of 60 V. They employed a titanium foil with a thickness of 0.25 mm, immersed in a solution comprising 0.3 wt% NH_4_F and 2% H_2_O in ethylene glycol. Our results—derived from anodization in a solution comprising 0.7 wt% NH_4_F and 3.5 wt% distilled water at 30 V—revealed the length of the TNT nanotubes to be 133 nm at the nanoscale. The SEM images also demonstrate that the obtained nanotubes are ordered and have clear open ends. Despite the low voltage (30 V), we compensated for this by increasing the concentrations of NH_4_F and H_2_O in the anode solution. Given the higher mass percentage of NH_4_F, compensation is accomplished by increasing the concentration of H_2_O, resulting in faster growth and, hence, longer nanotube lengths.

**Figure 3:**
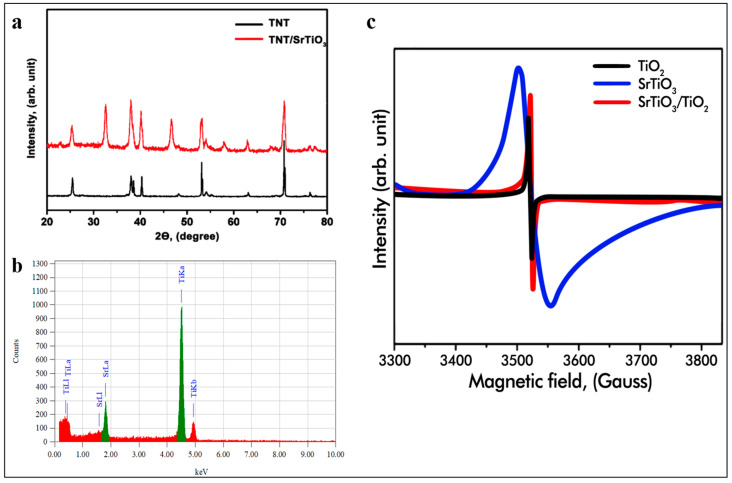
(**a**) X-ray diffraction analysis of combined TNT@ SrTiO_3_; (**b**) semi-quantitative elemental analysis of TNT@SrTiO_3_ particles; (**c**) EPR spectra of pristine TiO_2_ and SrTiO_3_ and pristine SrTiO_3_/TiO_2_ nanotube arrays after hydrothermal reaction of 5 h duration.

XRD analysis of the TNT@SrTiO_3_ samples was performed on an X-ray diffractometer with detection unit rotation angles ranging from 20° to 80° and a minimum detection unit movement step of 0.01, as shown in Figure 3a. The characteristic peaks of the TNT samples appear at 2θ 25.4°, 37.9°, and 53.4°, 71.5°, indicating the polycrystalline structure of the anatase, in good agreement with the standard map for TNT (JCPDS map 1286) [20]. In addition, the appearance of new peaks at 32.2°, 46.9°, and 57.8° in the X-ray diffraction spectrum of the TNT@SrTiO_3_ samples indicates the combination of two components in the composite, which additionally proves the successful connection and interaction between the components. This confirmation is based on a comparison of the diffraction spectra of the composite with TNT, which makes it possible to determine whether changes have occurred in the crystal structure during their combination. It is particularly important to note that the peak at 2θ, equal to 71.23°, has a high intensity, indicating the high crystallinity of the semiconductor. This is significant because the transport efficiency of charged carriers generated during photogeneration can be strongly dependent on the crystallinity of the material. Low crystallinity can lead to the inefficient migration of charged particles. In addition, semi-quantitative elemental analysis of the particles confirmed the composition of the obtained samples. The presence of the elements Ti and Sr was confirmed without detecting other impurities. According to the atomic percentages in the index (Figure 3b), it can be established that Ti/Sr is 81.10%/18.90%, respectively. These results confirm that the designs contain the expected elements and have no significant impurities.

**Figure 4:**
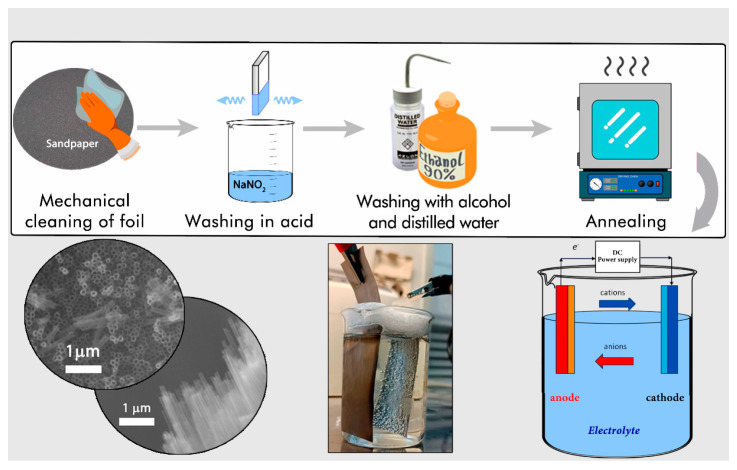
Schematic illustration of the stages of obtaining TNT.

Low-temperature electron paramagnetic resonance (EPR) spectra were determined on the SrTiO_3_/TiO_2_ samples to confirm the presence of Ti^3+^ and oxygen vacancies. The initial SrTiO_3_/TiO_2_ (Figure 4c, marked in red), containing mainly Ti^4+^ 3d0 states, exhibits a weak EPR signal, which may be due to the surface adsorption of O_2_ from air. For SrTiO_3_ and TiO_2_, a strong signal from Ti^3+^ spins (marked in blue and black) is also observed. It is generally believed that photoelectrons can be captured by Ti^4+^ and lead to the reduction of Ti^4+^ cations to the Ti^3+^ state, which is usually accompanied by the loss of oxygen from the surface of TiO_2_ and SrTiO_3_. Thus, these data clearly confirm that Ti^3+^ and oxygen vacancies were formed in all SrTiO_3_/TiO_2_, TiO_2_, and SrTiO_3_ samples.

TiO_2_ nanotubes can be produced in various ways [29], among which, the most widely studied is the use of electrochemical anodization. The advantage of anodic TiO_2_ nanotubes over TiO_2_ nanotubes produced by other methods is their availability and cost-effectiveness. Also, one of the advantages of this method is that the anodic TiO_2_ nanotubes grow vertically on the Ti substrate with nanotube holes on top and closed nanotube bottoms attached to the Ti substrate. Thus, no further immobilization on the substrate is required. The TNT layers are highly ordered, which favors a direct diffusion pathway. In addition, the nanotube layers can be removed from the Ti substrate and used as powders if required. Another advantage is that the nanotube layer thickness and nanotube diameter can be controlled by adjusting the anodization electrolyte, potential, and time [30].

## 3. Materials and Methods

### 3.1. Materials

Ti foil (99.9%; thickness, 0.1 mm; China), ethanol (45%), ethylene glycol (99.9%, Russia), ammonium fluoride, and sodium nitrate (70%) were used without further purification. Distilled water was used as a solvent in all experiments.

### 3.2. Synthesis of SrTiO_3_

SrTiO_3_ was obtained using a chemical precipitation method [24,31,32,33]. For this purpose, 2.54 g of Sr (NO_3_)_2_ was mixed with 100 mL of distilled water; then, 0.958 g of TiO_2_ was added in a 1:1 ratio of Ti and SrTiO_3_ to this solution. The solution was then treated for 30 min in an ultrasonic bath. The solution was gradually added while maintaining vigorous stirring, and the pH of the mixture was brought to 6–7 using 10% NH_3_OH solution. The suspension was washed several times with distilled water. The resulting powder was dried at 60 °C overnight and then calcined at 900 °C for 1 h.

### 3.3. Nanotube Synthesis

TiO_2_ was obtained using an anodization method. The 0.1 mm thick Ti foil was initially cut into 1 cm × 6 cm samples and mechanically polished with P150 sandpaper. The sheets were then ultrasonically treated in sodium nitrate, ethanol, and distilled water for final cleaning. Electrochemical anodization experiments were carried out in a two-electrode electrochemical cell, where titanium foil served as the working electrode and a sheet of nickel foil as the counter electrode at constant potential and room temperature (≈22 °C). Figure 1 shows a schematic of the titanium nanotube formation process. A constant current power supply unit model, UNI-T UTP3315TPL from UNI-TREND Technology, China, was used. This unit was used as a voltage source to control the anodization. The electrolyte for anodizing consisted of ethylene glycol with 0.7 wt% NH_4_F and 3.5 wt% distilled water added. The anodization process was carried out at 30 V for 96 **h** at room temperature. The anodized titanium nanotube samples were then placed in ethylene glycol and subjected to ultrasonic stirring until the nanotube film separated from the titanium substrate. The suspension was filtered; the residue was washed several times with distilled water. The resulting powder was dried at 60 °C for 3 h and then calcined at 450 °C for 1 h.

### 3.4. Synthesis of TNT@SrTiO_3_

To create the combined TNT@ SrTiO_3_ structure, powders of 0.2 g of TNT and 0.1 g of SrTiO_3_ were taken, mixed with 40 mL of distilled water, and placed in a stainless autoclave. The sealed autoclave was heated to 90 °C and incubated for 6 h. At the end of the experiment, the autoclave was cooled to room temperature. The samples were then washed with distilled water and dried in an oven for 5 h at 60 °C.

### 3.5. Material Characterization Techniques

The morphologies of the TNT and the combined TNT@SrTiO_3_ were analyzed using a JSM-6490LA scanning electron microscope from JEOL, Tokyo, Japan. TESCAN MAIA3 XMU scanning transmission electron microscopy (STEM) was used to further investigate the morphology at high resolution. The crystal structure of the samples was studied using a Drone-8 X-ray diffractometer. An EPR spectrometer “JEOL” (JES-FA200, Japan) was also used. Measurements were in ranges of ~9.4 GHz (X-Band) and ~35 GHz (Q-Band). Microwave frequency stability—~10^− 6^. Sensitivity—7 × 109/10^− 4^ Tl. Resolution—2.35 μT. Output power—from 200 mW to 0.1 μW. Quality factor (Q-factor)—18,000.

## 4. Conclusions

In this paper, the synthesis of arrays of TiO_2_ nanotubes using an electrochemical anodization method was successfully demonstrated. The obtained nanotubes have clear and open ends and are 133.9 nm long, and their membranes are more than 1 μm thick. The anodization process of 0.1 mm thick Ti foil at 450 °C can easily produce such nanotube arrays. SEM analysis showed that the TNTs are characterized by clear and open tube ends, with an average outer diameter of 1 μm and an inner diameter of 69 nm, and their length is 133 nm. In addition, a combined structure of TNT@SrTiO_3_ was fabricated in this study using chemical autoclave synthesis techniques. X-ray phase analysis confirmed the high crystallinity and orientation of crystallites along the preferential growth direction, indicating the successful formation of the structure. The results obtained here have potential significance for various fields including the sunlight-induced photocatalytic decomposition of water and other applications in energy conversion and storage. Further research and development in this area can contribute to the development of innovative technologies and improve the efficiency of energy systems.

## Data Availability

The data are contained within the article.
